# The role of private health providers in HIV testing: analysis of data from 18 countries

**DOI:** 10.1186/1475-9276-13-36

**Published:** 2014-05-12

**Authors:** Doug Johnson, Xi Cheng

**Affiliations:** 1 International Health Division, Abt Associates Inc., 4550 Montgomery Ave #800N, Bethesda, MD, USA; 2 Department of Health Services Policy and Management, Arnold School of Public Health, University of South Carolina, 915 Greene Street, Columbia, SC 29208, USA

**Keywords:** HTC, HIV/AIDS, Private health sector

## Abstract

**Introduction:**

HIV testing and counseling is a critical component of the overall response to the HIV epidemic in low and middle income countries. To date, little attention has been paid to the role of private for-profit providers in HIV testing.

**Methods:**

We use data from Demographic and Health Surveys and AIDS Indicators Surveys to explore the extent to which this sector provides HIV testing in 18 developing countries.

**Results:**

We find that use of the private sector for HIV testing varies significantly by country, with private for-profit providers playing a significant role in some countries and a relatively minor one in others. At the country level, use of private providers for HIV testing is correlated with use of private providers for other health services yet, in many countries, significant differences between use of the private sector for HIV testing and other services exist. Within countries, we find that wealth is strongly associated with use of the private sector for HIV testing in most countries, but the relative socio-economic profile of clients who receive an HIV test from a private provider varies considerably across countries. On the one measure of quality to which we have access, reported adherence to antenatal care testing guidelines, there are no statistically significant differences in performance between public and private for-profit providers in most countries after controlling for wealth.

**Conclusions:**

These results suggest that strategies for supervising and engaging private health providers with regard to HIV testing should be country specific and take into account local context.

## Introduction

HIV testing and counseling (HTC) is a critical component of the overall response to the HIV pandemic in developing countries. While numerous studies have examined the role of public health providers in providing HIV counseling and testing, there is a paucity of data on the role played by private providers in HIV testing in developing countries [1-3]. In most developing countries, supervision of private providers is weak and reporting by private providers to public agencies limited and inconsistent. Thus, even basic descriptive statistics on the extent of use of private providers for HIV testing are rarely included in national health statistics [4].

Better understanding the role of the private sector in HTC is important for two reasons. First, in many developing countries, a large share of the population, even the poor, access health care from the private sector [5]. Given the high use of the private sector for health care in many countries, governments should ensure that private health providers are offering HIV tests in cases where testing is indicated (or referring patients to another facility where testing may be conducted), adhering to national HTC guidelines, and referring HIV-positive patients to prevention, care and treatment services. Second, relying solely on HTC statistics from the public sector may lead to under-reporting of national testing rates, particularly among groups with high utilization of private providers for testing.

In this paper, we analyze the role played by private commercial health providers in providing HIV testing in 18 countries. This analysis builds on previous analysis by Wang et al. [5] which looked at use of the private sector for HIV testing in 12 countries and analyzed the relationship between wealth and use of the private sector for testing. Our research extends this analysis by using several newly available datasets and exploring additional lines of investigation. We estimate the overall proportion of those tested for HIV who received their test from a private provider in each of these countries, compare use of the private sector for HIV testing with use of the private sector for other health services, and investigate the relationship between household wealth and use of the private sector for HIV testing.

In addition, we compare public and private providers on the one measure of quality for which we have data: adherence to HIV testing protocols during antenatal care (ANC) visits. According to the World Health Organization’s (WHO) provider-initiated testing and counseling guidelines, all women receiving ANC should be offered an HIV test even in countries with concentrated and low-level HIV epidemics [6]. In all countries for which analysis was performed, with the possible exception of Benin, Guyana, Haiti, and Malawi, official national guidelines echo this recommendation that all women receiving ANC be offered an HIV test^a^. (In the cases of Benin, Guyana, Haiti, and Malawi, we were unable to determine the official policy regarding HTC during ANC).

## Methods

### Country selection

This report uses data from the Demographic and Health Surveys (DHS) and the AIDS Indictors Surveys (AIS) conducted by the USAID-funded Measure DHS project. DHS and AIS are large, nationally- representative, household surveys focused on maternal and child health in general (in the case of DHS) and HIV/AIDS in particular (in the case of AIS)^b^. The questionnaires, sampling strategies, and other methods used in the DHS and AIS surveys are very similar across countries. Further, questions included in AIS are largely a subset of questions included in the DHS and thus data from the two sets of surveys are comparable.

The DHS and AIS began including questions on the source of HIV testing in 2004, and surveys with such questions have been conducted in 21 countries since then. Three countries have been excluded from this analysis as too few respondents had received an HIV test (Guinea 2005 and Chad 2004) or the non-response rate to the question regarding source of HIV testing was too high (Rwanda 2005). In the remaining 18 countries, we used the latest dataset available if more than one dataset included data on source of HIV test. Out of the 18 countries for which we have data, 13 are in Sub-Saharan Africa (Benin, Cote D'Ivoire, Ethiopia, Kenya, Lesotho, Liberia, Malawi, Mozambique, Sierra Leone, Swaziland, Tanzania, Uganda, and Zimbabwe), two are in Southeast Asia (Cambodia and Vietnam), and three are in the Caribbean region (Dominican Republic, Guyana, and Haiti). With the exception of the Dominican Republic, Cambodia, and Vietnam, all countries have HIV prevalence rates over 1%. Sample size and other key statistics for each country included in this analysis are provided in Table 1.

**Table 1:** Datasets used in the analysis

**Country**	**Year**	**Dataset type**	**GDP per capita (2012 USD)**	**National HIV prevalence**	**Total women in sample**	**#Women tested for HIV in sample**	**Total men in sample**	**#Men tested for HIV in sample**
Benin	2006	DHS	602	1.2%	17,794	3,055	5,321	676
Cambodia	2010	DHS	795	0.5%	18,754	4,695	8,239	1,986
Cote D'Ivoire	2005	AIS	908	4.8%	5,183	397	4,503	295
DR	2007	DHS	4,334	0.8%	27,195	18,104	27,975	11,651
Ethiopia	2011	DHS	358	1.5%	16,515	6,915	14,110	5,991
Guyana	2008	DHS	2,558	1.2%	4,996	2,525	3,522	1,272
Haiti	2006	DHS	515	2.1%	10,757	1,918	4,958	508
Kenya	2008	DHS	794	6.3%	8,444	4,915	3,465	1,437
Lesotho	2008	DHS	764	23.6%	7,624	5,193	3,317	1,321
Liberia	2007	DHS	211	1.8%	7,092	287	6,009	346
Malawi	2010	DHS	339	11.0%	23,020	17,046	7,175	3,845
Mozambique	2009	AIS	428	11.5%	6,413	2,596	4,799	1,073
Sierra Leone	2008	DHS	348	1.6%	7,374	1,006	3,280	278
Swaziland	2006	DHS	2,894	25.7%	4,987	2,040	4,156	804
Tanzania	2010	DHS	524	5.6%	10,139	5,732	2,527	1,055
Uganda	2011	DHS	487	6.5%	8,674	6,505	2,295	1,282
Vietnam	2006	AIS	731	0.4%	7,289	585	6,707	603
Zimbabwe	2011	DHS	776	14.3%	8,948	5,499	7,480	2,810

Notes: All data on GDP per capita and HIV prevalence from World Bank data catalogue (accessed from http://data.worldbank.org) except for data on HIV prevalence in Ethiopia which was obtained from 2012 UNAIDS Ethiopia country progress report (access from http://www.unaids.org/en/dataanalysis/knowyourresponse/countryprogressreports/2012countries/GAP%20Report%202012.pdf).

### Variables

Our analysis focuses on the use of private for-profit providers (hereafter referred to as private providers) for HIV testing. We define “private providers” as providers who are not government employees and who seek to earn a profit. A challenge faced in performing this analysis was identifying which types of providers are private and which are not. Answer choices for the question regarding source of most recent HIV test included both government and non-government facilities but did not explicitly distinguish between private commercial providers and other non-governmental providers. In addition, the response options to this question vary across countries, with specific local options included. In most cases, determining whether an answer option referred to a private commercial provider was straightforward. However, in cases where it was unclear, we relied on local experts for guidance in coding the source of HIV test^c^. Non-response rates for the question regarding provider type for most recent HIV test are below 5% for all datasets except for the Malawi men’s dataset in which the non-response rate was 8.5%.

We utilize the wealth quintile variable included in the DHS and AIS datasets to analyze testing by socio-economic status. As DHS and AIS surveys do not include questions on consumption expenditure, the wealth index is calculated using information on asset ownership and housing characteristics. Wealth rankings based on this index have been shown to be broadly consistent with wealth rankings based on consumption expenditure aggregates [7]^d^.

In the second section of this paper, we compare use of the private sector for HIV testing with use of the private sector for other health services by country. DHS datasets include data on source of care for five additional health services: STI treatment, family planning, child fever/cough, ANC, and child diarrhea. AIS datasets do not include this data and countries for which we rely on AIS datasets are excluded from this analysis.

The DHS and AIS datasets do not provide information on the overall quality of HIV testing and counseling provided by public and private practitioners but do provide information on whether HIV testing was provided during ANC. In particular, for women who gave birth in the five years prior to the survey and received antenatal care, DHS and AIS surveys ask whether the respondent was offered an HIV test, whether she received an HIV test, and whether she received the results from the test. This information was combined with information on the type of provider delivering the ANC to determine the proportion of public and private providers who offer HIV testing during ANC.

All analysis was performed using Stata 12.

### Statistical analysis

The majority of the results presented in this paper were compiled through basic tabulations and cross tabulations. Where appropriate, we have included confidence intervals (at the 95% level) of estimates and significance levels for comparisons of means based on t-tests.

In our comparison of use of the private sector for HIV testing with use of the private sector for other health services, we calculate correlation coefficients between overall utilization of the private sector for HIV testing and overall utilization of the private sector for other health services at the country level. (As the subset of respondents utilizing each service varies considerably we are unable to calculate the correlation at the individual level).

To compare adherence of public and private providers to ANC HIV testing guidelines, we employ a linear probability model to estimate the difference between public and private providers in the probability of offering of an HIV test after controlling for respondent wealth. Limiting the sample to women who were pregnant in the last five years and received ANC from either a public or private provider, the dependent variable is a binary variable for whether the respondent was offered an HIV test during ANC and our explanatory variables are a binary variable for whether the provider was from the private sector and binary variables for wealth quintiles. Use of a linear probability model facilitates easy interpretation of our results: the coefficient on the binary variable indicating use of a private provider is the expected increase in the probability of being offered an HIV test associated with receiving ANC from a private provider as opposed to a public provider^e^.

## Results

### Use of private for-profit providers for HIV testing by country

**Figure 1:**
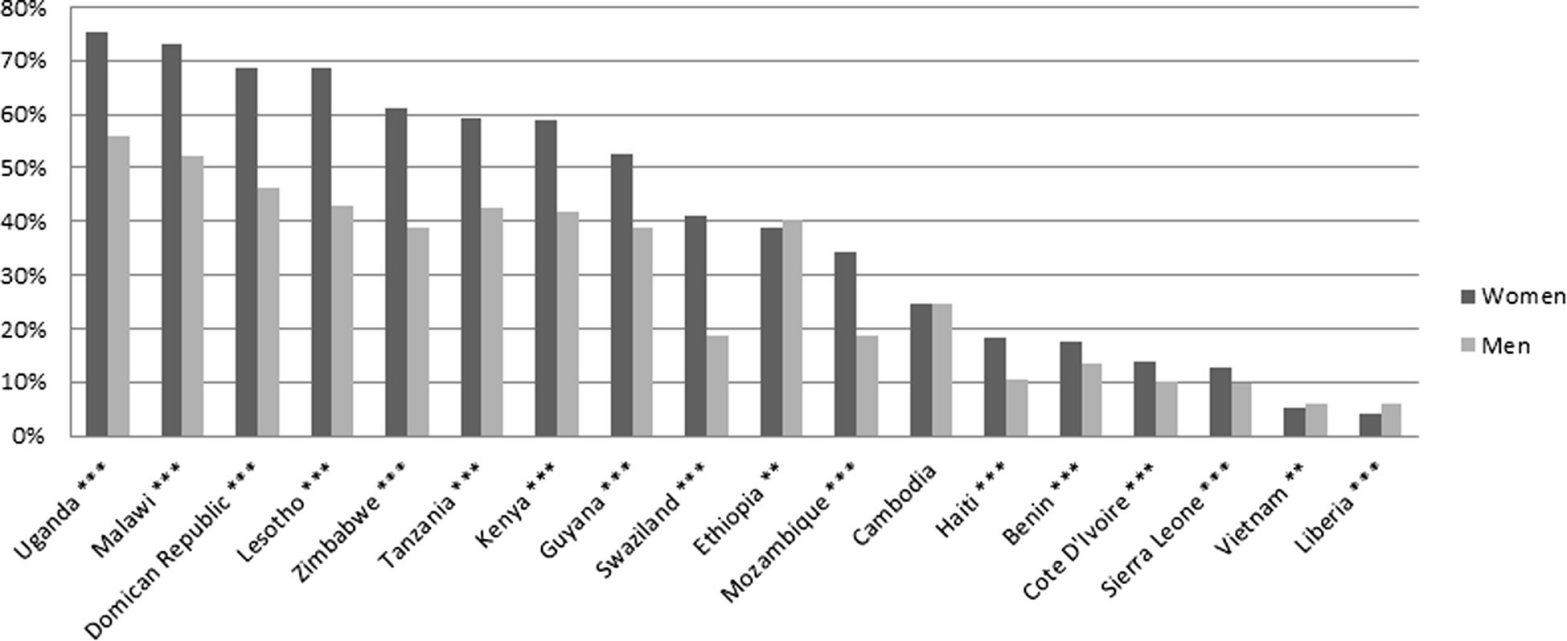
**Proportion of adults who have ever received an HIV test.** Note: Stars indicate statistical significance of *t* test of difference between men and women at the 10% (*), 5% (**), and 1% (***) levels.

**Figure 2:**
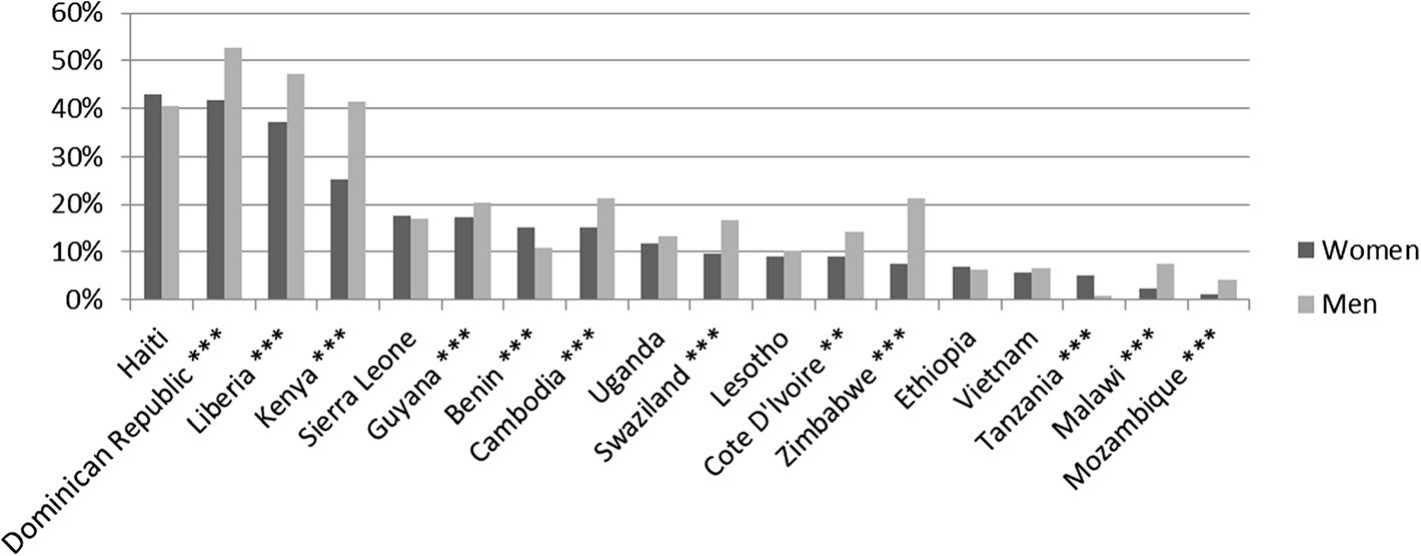
**Proportion of those tested for HIV who received test from private provider.** Note: Stars indicate statistical significance of *t* test of difference between men and women at the 10% (*), 5% (**), and 1% (***) levels.

Use of the private sector for HIV testing varies significantly by country with private providers playing a substantial role in some countries and a relatively minor one in others. Figure 1 displays the overall proportion of men and women who have ever received an HIV test by country. Figure 2 presents the proportion of men and women who were tested for HIV who received this test from a private provider. In Mozambique, only 1% of women who were tested for HIV received that test from a private provider, while in Haiti the comparable figure was 43%. Across countries, average utilization of the private sector for HIV testing was 15.6%.

A second salient result apparent from Figure 2 is that relative use of the private sector for HIV testing is higher among men than women in most countries. In 10 out of 18 countries, the difference between men and women’s utilization of the private sector for HIV testing was positive and statistically significant at the 5% level or higher. In only two countries, Benin and Tanzania, was this difference negative and statistically significant at the 5% level. In some countries, this difference is substantial. In Kenya for example, men who were tested for HIV were 16 percentage points more likely to have received that test from a private provider than women. These differences are partially, but not fully, explained by HIV testing at ANC. As the subsequent analysis shows, in most countries women are more likely to have received their most recent ANC care at a public facility than to have received their most recent HIV test from a public provider. Since testing during ANC accounts for a significant portion of total HIV testing among women, testing during ANC drives the overall figure for utilization of the private sector for HIV testing down^f^. Yet even after excluding women who received their most recent HIV test as part of ANC, men are still more likely to have been tested by a private provider than women. For those countries for which we have data on whether the most recent HIV test was during ANC, the average of the country-level differences between men and women including all women is 4.2 percentage points. If we exclude women who received their most recent HIV test during ANC, the average of the differences is 3.4 percentage points.

The relatively limited number of countries for which we have data prevents us from determining which country-level variables are most associated with high use of the private sector for HIV testing.

### Use of private providers for HIV testing compared to use of private providers for other health services by country

**Figure 3:**
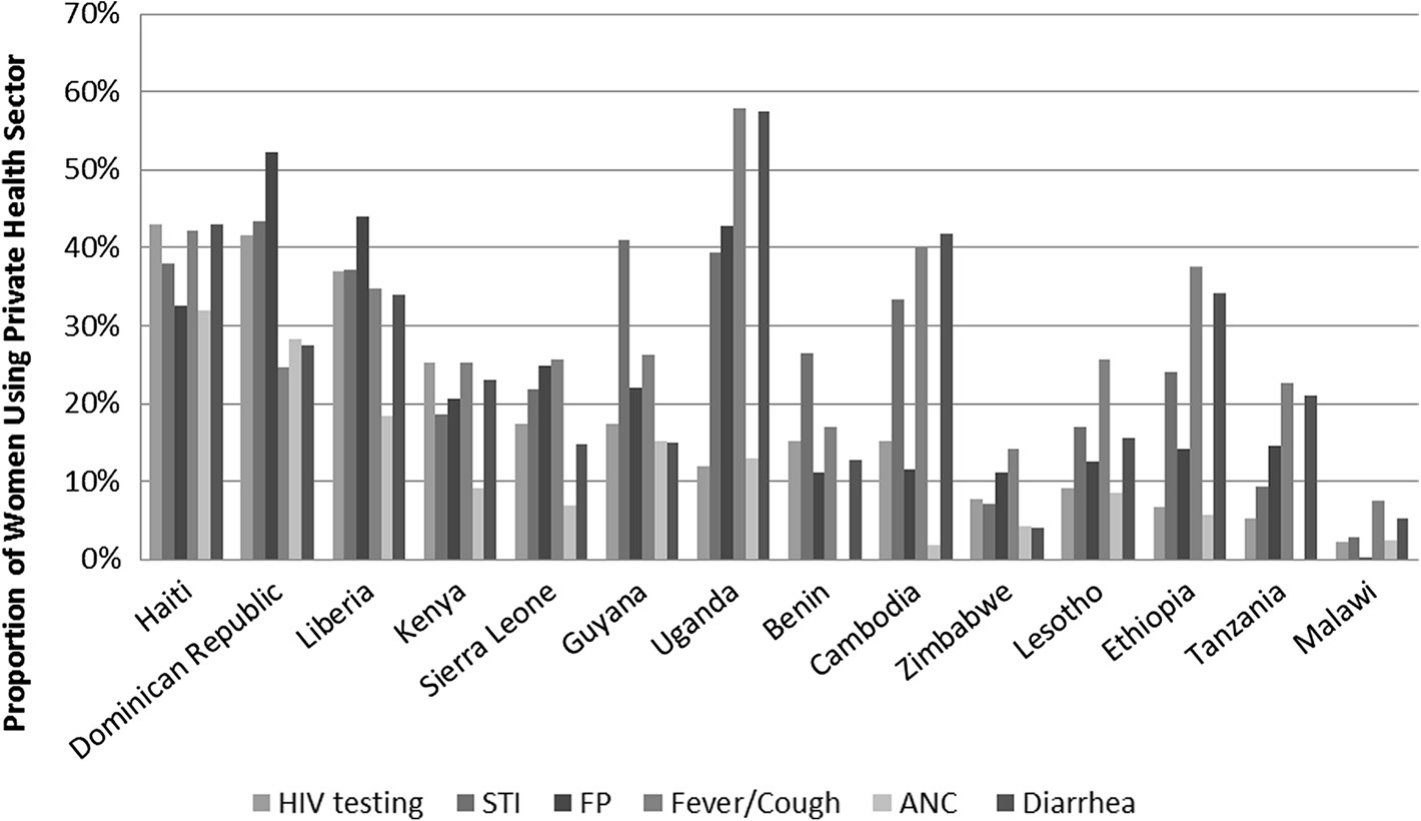
**Usage of private sector for various health services among women.** Note: Cote D’Ivoire, Mozambique, Vietnam, and Swaziland not included as data on source of other health services not included in these datasets. Data on provider type for ANC missing for Benin and Tanzania.

Comparing use of the private sector for HIV testing with use of the private sector for other health services may yield insight into the determinants of overall use of the private sector for HIV testing. If use of the private sector for HIV testing is highly correlated with use of the private sector for other services, then use of the private sector for testing is likely determined largely by the overall size of the private sector in a given country. On the other hand, if no such correlation exists, we must look elsewhere to explain rates of private sector utilization for HIV testing.Figure 3 displays overall use of the private sector for a variety of basic health services, including HIV testing, by country. Across the countries for which we have data, utilization of the private sector for HIV testing exhibits the strongest correlation with utilization of the private sector for ANC (correlation coefficient of .88, p-value of 0.0001). The correlation between utilization of the private sector for HIV and utilization of the private sector for family planning and STI treatment is also strong and statistically significant (correlation coefficients of .77 and .69 and p-values of .0014 and .0064 respectively). The correlation with utilization of the private sector for fever/cough and diarrhea is not statistically significant.

Despite high overall country level correlation between use of the private sector for HIV testing and other health services, significant differences between use of the private sector for these services exist. For example, in the East African countries of Uganda, Ethiopia, and Tanzania and in Cambodia, the private sector was used for HIV testing much less than for most other health services.

### Household wealth and use of the private sector for HIV testing

**Figure 4:**
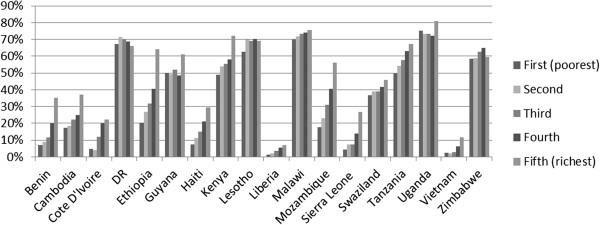
Proportion of women tested for HIV by wealth quintile.

**Figure 5:**
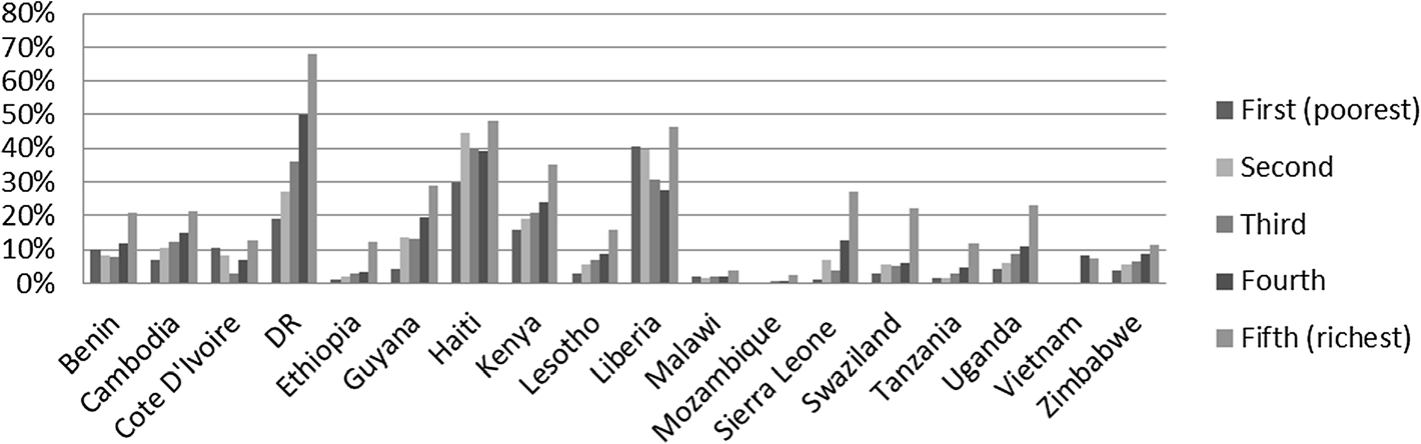
Proportion of women tested who received test from private provider by wealth quintile.

**Figure 6:**
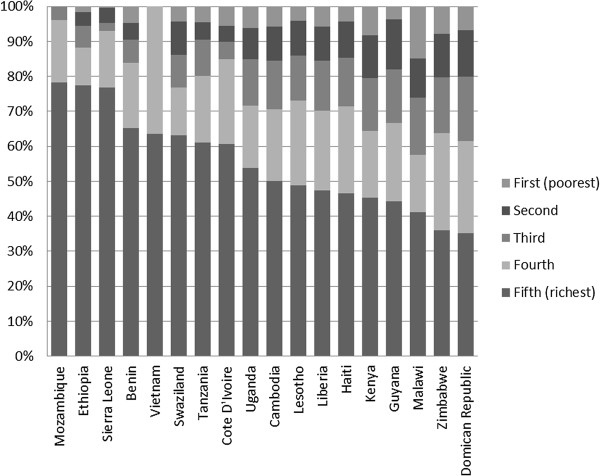
Proportion of women from each wealth quintile among those tested for HIV by a private provider.

Household wealth is strongly associated with being tested for HIV and, among those tested, use of private providers for HIV testing in most countries. Figure 4 displays HIV testing rates for women by wealth quintile. Figure 5 displays the proportion of women who received their most recent HIV test from a private provider among those tested. We do not present data on HIV prevalence rates by wealth quintile here, but note that other authors have found a positive correlation between wealth quintile and HIV prevalence in many of these countries [8]. In all countries but two (the Dominican Republic and Lesotho), the difference in HIV testing rates between women in the top two quintiles and other women is positive and statistically significant at the 1% level. Similarly, in all countries but four (Cote D’Ivoire, Haiti, Liberia, and Uganda), the difference in utilization of the private sector for HIV testing between women in the top two quintiles and other women is positive and statistically significant at the 1% level.As the wealthy are both more likely to be tested for HIV and, among those who are tested, more likely to be tested by a private provider, a large share of those tested by private providers in several countries come from the top wealth quintiles. Figure 6 presents the proportion of women from each quintile among those tested for HIV by a private provider. In 10 out of 18 countries, over half of women tested for HIV by a private provider are from the top wealth quintile.

The figure also shows that there is wide variation in the socio-economic profile of those tested by private providers for HIV across countries. Despite higher HIV testing rates and utilization of private providers for HIV testing among the wealthy, in several countries a substantial portion of those tested by private providers are from the lower wealth quintiles. In five of the countries, at least one third of women tested by a private provider were from one of the bottom 3 wealth quintiles.

### Comparing adherence of public and private providers to ANC HIV testing guidelines

**Figure 7:**
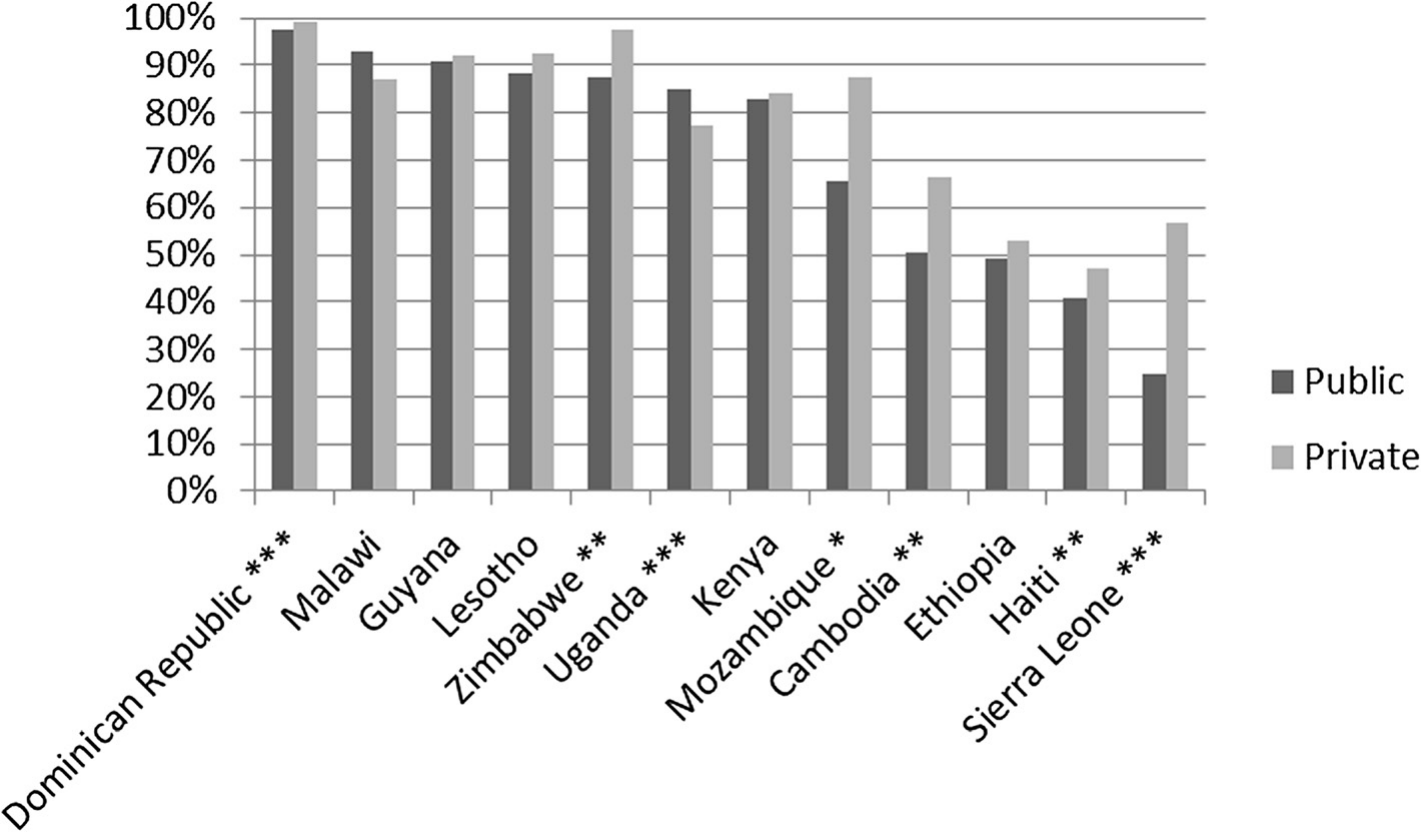
**Proportion of women who received ANC who were offered HIV test by provider type.** Note: Benin, Cote D’Ivoire, Liberia, Swaziland, Tanzania, and Vietnam excluded due to lack of data.

Figure 7 shows the proportion of women who reported that they were offered an HIV test during ANC disaggregated by provider type^g^. In 5 out of 12 countries for which there are data, the difference between the proportion of women who were offered an HIV test during an ANC visit at a private provider and the proportion of women who were offered an HIV test during an ANC visit to a public provider is positive and statistically significant at the 5% level. In only one country, Uganda, was this difference negative and statistically significant at the 5% level.

This overall comparison of public vs. private providers ignores important potential differences in the types of patients who visit public and private providers for ANC. For example, patients who receive ANC care from private providers may be wealthier on average and thus more likely to demand, and be able to afford, an HIV test. We attempt to isolate the effect of provider type on the likelihood of being offered an HIV test during ANC by performing linear regressions, for each country, of whether the respondent was offered an HIV test on a binary variable indicating whether the provider was from the private sector and binary variables for wealth quintiles. Table 2 presents results from these regressions. After controlling for wealth, the difference in reported rates of being offered an HIV test during ANC between private and public providers is still positive and statistically significant at the 5% level or higher in 3 countries -- the Dominican Republic, Sierra Leone, and Zimbabwe – but is negative and statistically significant at the 10% level in 2 countries -- Malawi and Uganda.

**Table 2:** Combined results from regressions of whether respondent was offered HIV test during ANC on provider type and wealth quintile

**Country**	**Coeff on binary variable for private provider (percentage points)**	**Standard error (percentage points)**	**N**
Cambodia	5.62	(7.37)	3498
DR	1.24**	(0.57)	5863
Ethiopia	−7.59	(5.77)	2151
Guyana	−2.06	(2.82)	1030
Haiti	2.48	(2.72)	2214
Kenya	−2.86	(3.36)	3014
Lesotho	1.04	(2.94)	1438
Malawi	−6.48*	(3.35)	9720
Mozambique	7.05	(10.71)	1967
Sierra Leone	14.07***	(3.98)	1971
Uganda	−10.31***	(2.83)	3458
Zimbabwe	4.19**	(1.90)	2638

Notes: Stars indicate statistical significance of the coefficient at the 10% (*), 5% (**), and 1% (***) levels.

## Discussion

Using data from 18 countries, we analyzed overall patterns in use of the private sector for HIV testing. Our results expand and update the results from Wang et al. [5] who analyzed use of the private sector for HIV testing for a smaller set of countries and performed a more limited range of analyses. Our analysis shows that use of the private sector for HIV testing varies substantially across countries, with private providers playing a prominent role in some countries and a relatively minor one in others. For example, in Haiti 43% of women tested for HIV received their most recent test from a private provider, while in Mozambique the comparable figure was only 1%. Similarly, we find that while in most countries wealthier women are both more likely to be tested for HIV and more likely to use a private provider if they are tested, the socio-economic profile of women tested for HIV by private providers also varies considerably across countries. This result contrasts with other recent studies, such as World Bank Staff [4] which, using DHS data on treatment for child diarrhea in sub-Saharan Africa, found relatively little variation in utilization of private providers by wealth quintile.

These results suggest that strategies for supervising and engaging private health providers should be country specific and take into account local context. A strategy for engaging private providers appropriate for countries with very low private sector utilization for HIV testing and in which users of private providers for HIV testing are predominantly better off, such as Mozambique or Vietnam, may not be suitable for countries with high utilization and more equitable use of private providers for HIV testing such as Haiti, Liberia, and the Dominican Republic.

In the set of analyses, we show that, at the country level, utilization of the private sector for HIV testing is strongly correlated with utilization of the private sector for ANC, STI treatment, and family planning but not particularly correlated with utilization of the private sector for treatment of childhood illness such as fever and diarrhea. This relationship may reflect differences in the types of providers women seek out for personal health issues versus those of their children. Alternatively, it may reflect differences in the provider skills and infrastructure required to perform HIV tests compared to these other services.

Closer inspection of utilization of the private sector for these health services by country reveals that while there is strong overall correlation across countries in utilization, in several countries use of the private sector for HIV testing lags behind use of the private sector for other health services. In particular, in the East African countries of Uganda, Ethiopia, and Tanzania and in Cambodia, the private sector was used for HIV testing far less than for most other health services. Differences in private sector utilization may be due to differences in levels of public and donor funding for HIV testing versus other health services or a variety of other factors. The limited number of countries for which we have data prevents us from determining whether these differences are correlated with country level variables. Further research at the country level to determine reasons for these differences is warranted.

We find that men use private providers for HIV testing at significantly higher rates than women in several countries. HIV testing during ANC visits helps explain a portion of this difference. Yet even after accounting for HIV testing during ANC in the countries for which we have data, men are still more likely to use a private provider for HIV testing.

An important concern for policymakers charged with oversight of the health sector is whether the private sector delivers care at the same level of quality as the public sector. Some researchers and policymakers have questioned whether the quality of care delivered by private health providers is as good as that delivered by public providers. For example, in a meta-review of research comparing quality of public and private health providers, Basu et al. [9] show that several of the studies they reviewed found that quality of service provision by private providers was lower than that of public providers. We compare public and private on the one measure of quality to which we have access – reported adherence to international ANC testing protocols. While HIV testing during ANC captures only a small part of overall quality of HIV testing, it is a particularly useful metric with which to compare public and private providers. According to the WHO, HIV testing should always be performed during ANC regardless of circumstances [6]. Further, DHS data reveals that testing during ANC accounts for a large share of total HIV testing among women in many countries. Without controlling for wealth, we find that reported adherence to HIV testing protocol is higher for private providers than public facilities in 5 countries and worse in 2. Controlling for wealth, the disparity in performance between public and private providers on ANC testing across countries, for the most part, disappears.

Our analysis suffers from several limitations. In most cases, distinguishing between private for profit providers and other types of providers based on the answer options included in the DHS questionnaires was straightforward. Yet in a small number of cases we were unsure whether an answer option referred to a private for profit provider, a private not-for-profit provider or to both types. In the second half of this paper, we compare public and private providers on adherence to ANC testing guidelines. While useful, this relatively crude comparison should not be interpreted as an overall comparison of the quality of HIV testing by public and private providers which we are unable to perform due to a lack of data on quality. Data on the relative quality of public and private providers, collected perhaps through mystery clients or vignette surveys, would have greatly strengthened our analysis. Lastly, we are unable to draw any conclusions as to why respondents chose one type of provider over another. Data on the availability of public and private facilities, which was included in previous versions of DHS surveys but subsequently dropped, would have allowed us to investigate what affect distance to different facility types had on use.

HIV counseling and testing is a critical part of the overall response to the pandemic. In this paper, we examine the role of the private sector in HIV testing. We show that the private sector, despite the relatively little attention it receives, plays a significant role in delivering HIV testing in several countries. Further research at the country level examining the policy context with regards to HIV testing conducted by private providers may shed additional light on how private providers can be further leveraged to increase HIV testing rates, and ensure a more accurate accounting of the epidemic through improved reporting of positive test cases.

## Endnotes

^a^For Cambodia, see http://www.who.int/hiv/pub/guidelines/cambodia_art.pdf?ua=1; for Cote D’Ivoire see http://www.pepfar.gov/pepfar/press/84152.htm; for Dominican Republic see http://www.who.int/hiv/pub/guidelines/dominican_art.pdf; for Ethiopia see http://www.who.int/hiv/pub/guidelines/ethiopia_art.pdf?ua=1; for Kenya see http://nascop.or.ke/library/HTC/National%20Guidelines%20for%20HTC%20in%20Kenya%202010.pdf; for Lesotho see http://www.who.int/hiv/pub/guidelines/lesotho_art.pdf; for Liberia see http://apps.who.int/medicinedocs/documents/s18366en/s18366en.pdf; for Mozambique see http://www.unicef.org/infobycountry/mozambique_58831.html; for Sierra Leone see http://www.whosierraleone.org/1_docs/mohspartnersdocs/sl_operational_plan.pdf; for Swaziland see http://www.who.int/hiv/pub/guidelines/swaziland_art.pdf; for Tanzania see http://www.who.int/hiv/pub/guidelines/tanzania_art.pdf and http://ihi.eprints.org/823/1/MoHSW.pdf_(53).pdf; for Uganda see http://www.who.int/hiv/pub/guidelines/uganda_art.pdf; for Vietnam see http://www.unicef.org/vietnam/MIV_Eng.pdf; for Zimbabwe see http://www.safaids.net/content/zimbabwe-national-hiv-and-aids-strategic-plan-znasp-ii-2011-%E2%80%93-2015

^b^Further details on the sampling strategy and methodology used for DHS and AIS surveys may be found at http://dhsprogram.com/What-We-Do/Survey-Types/DHS-Methodology.cfm and http://dhsprogram.com/What-We-Do/Survey-Types/AIS-Methodology.cfm

^c^Details of how we coded each answer option available from the authors on request.

^d^For more information on how wealth quintiles are calculated for DHS and AIS surveys see Rutstein and Johnson [10].

^e^Linear probability models have been criticized on the basis that they do not provide consistent estimates of the marginal effects if the true data generating process is non-linear, as it often is in the case of binary dependent variables. While valid, this criticism is equally applicable to non-linear models which do not accurately capture the true non-linear data generating process. Further, our model is nearly fully saturated (in the sense that our model includes separate parameters for almost all possible values of the explanatory variables) and thus unlikely to suffer from serious non-linearities. As a specification check, we count the number of predicted values from our model which fall outside the unit interval [11]. For all countries except the Dominican Republic all predicted values from our model fall within the unit interval. In the case of the Dominican Republic, less than 10% of the predicted values fall outside the unit interval.

^f^The proportion of women tested from HIV who received their most recent HIV test as part of ANC varies from 10.8% in Ethiopia to 47.6% in Benin. Data on proportion of women tested for HIV who received most recent test during ANC not available for Liberia, Swaziland, Tanzania, Mozambique, Vietnam, and Cote D’Ivoire.

^g^Results for Benin, Cote D’Ivoire, Liberia, Swaziland, Tanzania, and Vietnam excluded as these datasets do not include information on source of ANC care.

## Competing interests

The authors s declare that they have no competing interests.

## Authors’ contributions

DJ designed the study, performed data analysis, and wrote the manuscript. XC assisted in data analysis and reviewed the manuscript. Both authors read and approved the final manuscript.
